# OA33 Incidence of chronic recurrent multifocal osteomyelitis in the UK and Republic of Ireland: initial results from 13 months of surveillance study

**DOI:** 10.1093/rap/rkac066.033

**Published:** 2022-09-28

**Authors:** Chenqu Suo, Daphne Chia, Andoni Toms, Anish Sanghrajka, Athimalaipet Ramanan, Orla Killeen, Benjamin Jacobs, Cristina Ilea, Kamran Mahmood, Sandrine Compeyrot-Lacassagne, Kate Armon

**Affiliations:** Department of Paediatrics, University of Cambridge, Cambridge, United Kingdom; Department of Paediatrics, University of Cambridge, Cambridge, United Kingdom; Department of Radiology, Cambridge University Hospitals, Cambridge, United Kingdom; Department of Truama and Orthopaedics, Norfolk and Norwich University Hospitals, Norwich, United Kingdom; Department of Paediatric Rheumatology, Bristol Royal Hospital for Children, Bristol, United Kingdom; National Centre for Paediatric Rheumatology, Children's Health Ireland, Dublin, Ireland; Royal National Orthopaedic Hospital, London, United Kingdom; Royal National Orthopaedic Hospital, London, United Kingdom; Department of Rheumatology, Alder Hey Children's Hospital, United Kingdom; Great Ormond Street Hospital, London, United Kingdom; Department of Paediatrics, University of Cambridge, Cambridge, United Kingdom; Department of Paediatric Rheumatology, Cambridge University Hospitals, Cambridge, United Kingdom

## Abstract

**Introduction/Background:**

Chronic Recurrent Multifocal Osteomyelitis (CRMO), also known as chronic nonbacterial osteomyelitis (CNO), is a rare autoinflammatory condition affecting the bones. It occurs primarily in children and teenagers and is characterised by bone pain and swelling in the absence of infection or tumour. The incidence of CRMO remains uncertain, with estimates ranging from 0.4-1 per 100,000 person years.

**Description/Method:**

The primary aim of the study was to identify the incidence of CRMO in patients under the age of 16 in the United Kingdom (UK) and Republic of Ireland (ROI). Additional aims include describing the demographics, clinical features, treatment, and healthcare needs of patients with CRMO. A prospective surveillance study was undertaken via the British Paediatric Surveillance Unit. A monthly e-reporting card was sent to all registered paediatric consultants in the UK and ROI. A parallel surveillance study was sent via the British Society for Children’s Orthopaedics to identify patients managed solely by orthopaedics. A standardised questionnaire was sent to the reporting clinicians to collect further information.

**Discussion/Results:**

During initial 13 months of surveillance, 168 cases were reported. 23 questionnaires were not returned (13.7% of reported cases). After de-duplication, and removal of cases outside the reporting time period and age-group, 82 confirmed and 8 probable cases were included in these interim results. The estimated incidence of CRMO is 0.605 cases/100,000 children per year.

Median age at time of diagnosis was 10 years (range 3-16). 53 (58.9%) of cases were female. Median delay from symptom onset to diagnosis was 5 months and 16 patients (17.78%) had a delay of greater than 12 months. Most (48.9%) of the cases were diagnosed by paediatric rheumatology specialists. Other cases were diagnosed by orthopaedics (16.7%), general paediatricians (15.6%) or by a multidisciplinary team. 34 cases (37.8%) reported requiring hospital admission related to CRMO.

The most common presenting feature was bone pain (96.67%). 34 patients (37.8%) presented with clavicular pain, and thirty-one (34.4%) had unifocal bone pain. Patients also presented with bone swelling (52.2%), joint swelling (20.0%), fever (12.2%) and general malaise (13.3%). A median of 3 radiological investigations were reported for each case, of which 61 (67.7%) cases had whole body MRI performed. Additionally, 33 cases (36.67%) had bone biopsy. At initial reporting, the most common treatment was NSAIDs (90.0%) and bisphosphonates (33.3%).

**Key learning points/Conclusion:**

Our results estimate the incidence of CRMO as 0.605 cases per 100,000 person years. The study will continue to capture new CRMO cases for a further 12 months. Reported cases will be followed up for 24 months. This prospective study of all incident cases of CRMO within the UK and ROI will provide insight into the medium-term outcomes and treatment strategies used by clinicians. These results will provide a valuable baseline for further research and improvement in care for patients with CRMO.

